# Construction of prognostic signature of breast cancer based on N7-Methylguanosine-Related LncRNAs and prediction of immune response

**DOI:** 10.3389/fgene.2022.991162

**Published:** 2022-10-24

**Authors:** Jin Cao, Yichen Liang, J. Juan Gu, Yuxiang Huang, Buhai Wang

**Affiliations:** ^1^ Medical College, Yangzhou University, Yangzhou, Jiangsu, China; ^2^ Institute of Oncology, Northern Jiangsu People’s Hospital, Yangzhou, Jiangsu, China; ^3^ Department of Oncology, Northern Jiangsu People’s Hospital, Yangzhou, Jiangsu, China

**Keywords:** N7-methylguanosine (m7G), long non-coding RNA, breast cancer, prognostic model, immune response, the cancer genome atlas

## Abstract

**Background:** Long non-coding RNA (LncRNA) is a prognostic factor for malignancies, and N7-Methylguanosine (m7G) is crucial in the occurrence and progression of tumors. However, it has not been documented how well m7G-related LncRNAs predict the development of breast cancer (BC). This study aims to develop a predictive signature based on long non-coding RNAs (LncRNAs) associated with m7G to predict the prognosis of breast cancer patients.

**Methods:** The Cancer Genome Atlas (TCGA) database provided us with the RNA-seq data and matching clinical information of individuals with breast cancer. To identify the signature of N7-Methylguanosine-Related LncRNAs and create a prognostic model, we employed co-expression network analysis, least absolute shrinkage selection operator (LASSO) regression analysis, univariate Cox regression analysis, and multivariate Cox regression analysis. The signature was assessed using the Kaplan-Meier analysis and Receiver Operating Characteristic (ROC) curve. A nomogram and principal component analysis (PCA) were employed to confirm the predictive signature’s usefulness. Then, we examined the drug sensitivity between the two risk groups and utilized single-sample gene set enrichment analysis (ssGSEA) to investigate the association between predictive factors and the tumor immune microenvironment in high-risk and low-risk groups.

**Results:** Nine m7G-related LncRNAs (LINC01871, AP003469.4, Z68871.1, AC245297.3, EGOT, TFAP2A-AS1, AL136531.1, SEMA3B-AS1, AL606834.2) that are independently associated with the overall survival time (OS) of BC patients make up the signature we developed. For predicting 1-, 3-, and 5-year survival rates, the areas under the ROC curve (AUC) were 0.715, 0.724, and 0.726, respectively. The Kaplan-Meier analysis revealed that the prognosis of BC patients in the high-risk group was worse than that of those in the low-risk group. When compared to clinicopathological variables, multiple regression analysis demonstrated that risk score was a significant independent predictive factor for BC patients. The results of the ssGSEA study revealed a substantial correlation between the predictive traits and the BC patients’ immunological status, low-risk BC patients had more active immune systems, and they responded better to PD1/L1 immunotherapy.

**Conclusion:** The prognostic signature, which is based on m7G-related LncRNAs, can be utilized to inform patients’ customized treatment plans by independently predicting their prognosis and how well they would respond to immunotherapy.

## 1 Introduction

The most prevalent cancer in women worldwide is breast cancer, which accounts for 30% of all cancers in women globally and has a mortality-to-morbidity ratio of 15% (Siegel et al., 2020; Loibl et al., 2021). According to the status of the hormone receptors (ER and PR) and HER2 (ERBB2), breast cancer is categorized into four primary subtypes: Lumina-A, Lumina-B, HER2 positive, and triple negative breast cancer (TNBC) (Denkert et al., 2017), each subtype has a matching treatment. The main breast cancer treatments are surgery, radiotherapy, systemic chemotherapy, endocrine therapy, and targeted therapy (Krug and Loibl, 2018; Huang et al., 2021; Lau et al., 2022). While these treatments significantly increase the survival rate of BC patients, the disease still has a high mortality rate, particularly for triple-negative breast cancer, which will progress into a more invasive tumor form and have a poor prognosis (Aghili et al., 2013). Therefore, additional in-depth molecular level research is urgently required to identify novel therapeutic targets and direct clinically personalized BC treatment.

One of the most frequent base modifications in post-transcriptional regulation is N7-methylguanosine (m7G), which is added co-transcriptively to the 5 ′cap before the initial stage of transcription and other RNA processing activities (Song et al., 2020; Ming and Wang, 2022). The stability of transcripts, protein synthesis, and gene expression are all significantly influenced by N7-methylguanosine (m7G) capping (Furuichi et al., 1977). It has been discovered that m7G cap alteration can control practically all phases of the mRNA life cycle, including transcription, mRNA splicing, nuclear output, and translation (Muthukrishnan et al., 1975; Konarska et al., 1984; Lewis and Izaurralde, 1997; Pei and Shuman, 2002). The installation of the m7G modification at the five caps of mRNA is actively carried out by the enzyme RNA guanine-7 methyltransferase (RNMT) and its cofactor RNMT-activating miniprotein (RAM) (Trotman et al., 2017). Methyltransferase-Like 1 (METTL1), which binds to its matching cofactor WD repeat domain 4 (WDR4) and places the m7G alteration in tRNA, miRNA, and mRNA, is now the most researched m7G regulating factor (Alexandrov et al., 2002). Additionally, m7G is crucial for the onset and progression of numerous disorders; mutations in the m7G gene have been linked to teratoma, primitive dwarfism of microcephaly, and aberrant growth and differentiation (Shaheen et al., 2015; Lin et al., 2018; Deng et al., 2020). According to recent research, m7G may be linked to the onset and progression of cancer (Luo et al., 2022). According to Zhihang Chen et al., METTL1 influences translation and is reliant on the alteration of the m7G tRNA to induce hepatocellular cancer (Chen Z. et al., 2021). Jieyi Ma et al. have demonstrated that METTL1/WDR4-mediated modification of m7G tRNA and use of m7G codon promotes mRNA translation and lung cancer progression (Ma et al., 2021), Jie Ming et al. predicted the prognosis of renal cell carcinoma by building a prognostic model of m7G-related lncRNAs(Ming and Wang, 2022). In conclusion, m7G-related therapy is regarded as a novel anti-tumor therapeutic strategy that offers a fresh approach to the treatment of cancer.

The biological processes of cell proliferation and differentiation, genetic regulation of gene expression, RNA attenuation, RNA splicing, protein folding, and microRNA (miRNA) regulation all depend on long non-coding RNAs (LncRNAs), which are RNAs that cannot encode proteins and contain more than 200 nucleotides (Zhou et al., 2020; Fang et al., 2022). Recent research has demonstrated that lncRNAs play a role in the occurrence and progression of certain malignancies, including drug resistance, invasion, and proliferation (Ulitsky and Bartel, 2013). BinXu et al. found that (Bin et al., 2018) IncRNAs may have a variety of effects on the onset and progression of breast cancer, making them useful as indicators for the early detection, assessment, and prognosis of the disease. Yinan Wu et al. noted (Wu et al., 2019)that *via* controlling invasion, migration, epithelial-mesenchymal transformation (EMT), and metastasis, LncRNA has a role in fostering distant metastasis of breast cancer. By encouraging the localization and transcriptional inhibitory action of TRIM28, Alex J. Gooding et al. discovered that (Gooding et al., 2017) LncRNA BORG promotes the metastatic growth of potential breast cancer cells, resulting in the metastasis and recurrence of breast cancer. In order to predict the outcome of immunotherapy, Fangyue Chen et al. created a predictive profile of necroptosis-related lncRNAs in breast cancer (Chen et al., 2022). However, there are few studies based on m7G-related LncRNAs in breast cancer.

As a result, we developed the m7G-related lncRNA signature using the Cancer Genome Atlas (TCGA) database to assess the prognosis of BC patients. We also conducted the gene set enrichment analysis (GSEA) at the same time to investigate the potential mechanism, search for new biomarkers to predict the prognosis and immunotherapy response of BC patients, and offer a new method for precise treatment and tailored management of BC patients.

## 2 Methods and materials

### 2.1 Sample collection and screening

From the Cancer Genome Atlas (TCGA) database (https://portal.gdc.cancer.gov/), we were able to access the RNA-seq data and associated clinical information of patients with breast cancer. To lessen the statistical variance, patients with an OS of fewer than 30 days were eliminated from the clinical information screening. Supplementary Table S1 lists the clinicopathological features of BC patients.

### 2.2 Obtaining of lncRNAs and N7-Methylguanosine-Related genes

We acquired 152 genes associated with m7G from previously published articles (Ming and Wang, 2022). The Pearson correlation was used to determine the relationship between lncRNAs and genes associated with m7G. The screening criteria were |*R* ^2^ | > 0.4 and *p* < 0.001. Supplementary Table S2 provides the list of genes.

### 2.3 Analysis of the differences in N7-Methylguanosine-Related genes in BC

Analysis was done on the gene sets that differentiate paracancerous tissues from breast cancer tissues. As screening criteria, we employed |log 2 fold change (FC) > 1| and a false discovery rate (FDR) < 0.05 to identify differentially expressed genes associated with m7G (DEGs). The “pheatmap” and “ggpubr” packages in R software are used to develop volcano maps, heat maps, and block diagrams (Chen M. et al., 2021).

### 2.4 Building a predictive signature for m7G-Related lncRNAs in BC

We collected 1,090 matrix files of LncRNAs related to m7G. Then, lncRNAs associated with the prognosis of BC were found using univariate Cox regression analysis (*p* < 0.05). Then, to further screen m7G-related lncRNAs signature and build a prognostic model, multivariate Cox regression analysis and least absolute shrinkage selection operator (LASSO) regression analysis were used. The training set and testing set of the prognostic model created using Lasso regression are separated into two groups with a ratio of 1:1 (Ming and Wang, 2022). The m7G-related prognostic signature is created using the training set, and its accuracy is tested using the testing set. Table 1 shows the distribution of clinical characteristics in different cohorts. The following formula was used to determine the risk score: RiskScore is calculated as Coef LncRNAn×Expr LncRNAn, where Coef LncRNAn denotes the correlation between lncRNAs and patient survival in BC. The expression level of LncRNAs is represented by Expr lncRNAn. Each patient with BC has their risk score determined using this formula. Based on their median risk ratings, patients with breast cancer were divided into high-risk and low-risk categories (Fang et al., 2022).

**TABLE 1:** The clinical characteristics of patients in different cohorts.

Variables	TCGA dataset (n = 1,040)	Training set (n = 520)	Testing set (n = 520)
Age			
≤65	748	372	376
>65	292	148	144
Gender			
Female	1,028	515	513
Male	12	5	7
Stage			
I + II	769	386	383
III + IV	249	127	122
Unknow	22	7	15
T			
T1+T2	873	437	436
T3+T4	165	83	82
TX + Unknow	2	0	2
M			
M0	862	431	431
M1	21	9	12
MX + Unknow	157	80	77
N			
N0	486	245	241
N1	355	174	181
N2+N3	183	92	91
NX + Unknow	16	9	7

Abbreviations: N, lymph node; M, metastasis; T, tumor.

### 2.5 An evaluation of the prognostic value of lncRNAs related to m7G

The survival difference between high- and low-risk BC patients was then evaluated by using a survival curve, scatter plot, ROC curve, and heat map. To verify the predictive value of prognostic characteristics, two forest maps and ROC curves were used to show the results. The R software packages used in this process include “survival”, “survminer”, “timeROC”, “pheatmap".

### 2.6 Construction of nomogram and calibration curves

We combined the BC patient’s clinical variables to construct the nomogram and calibration curves of patients with BC for 1, 3, and 5 years. In this step we used the “rms” software packages.

### 2.7 KEGG and GO functional enrichment analysis

We explored the corresponding biological process by performing KEGG and GO functional enrichment analyses of m7G-related lncRNAs using GSEA 4.1.0 (http://www.broad.mit.edu/gsea/). The cutoffs for statistical significance were *p* < 0.05 and FDR <0.25 (Chen et al., 2022).

### 2.8 Relationship between immune-related variables and the predictive signature

Due to mounting evidence that immunological traits are crucial in the occurrence and growth of malignant tumors, we used ssGSEA to analyze the connection between risk score and immune-related factors and contrasted the immune checkpoint activation in patients with BC at low-risk and high-risk. The drug sensitivity of BC patients in the high- and low-risk groups was also examined.

### 2.9 Principal component analysis

Finally, we tested the risk model’s ability to distinguish between BC groups with high-risk and low-risk using principal component analysis (PCA). These procedures make use of the software packages “limma” and “scatterplot3d".

### 2.10 RNA extraction and quantitative real-time polymerase chain reaction

We collected blood samples from 10 cases of cervical cancer and normal tissues respectively from Northern Jiangsu People’s Hospital. Peripheral blood mononuclear cells (PBMCs) were extracted from each participant’s blood samples using Ficoll solution (Solarbio Life Sciences, Beijing, China). Using the RNA Extraction Kit, total RNA was extracted from PBMCs (Omega, Guangzhou, China). Using the PrimeScript RT Master Mix Kit, reverse transcription was carried out (Takara, Dalian, China). Relative gene expression was detected using the SYBR Premix Ex Tap kit (Accurate Biotechnology (Hunan) Co., Ltd., China). The relative expression levels of 9 m7G-related lncRNAs were calculated using the 2^−ΔΔCq^ method. Supplementary Table S3 contains primers.

### 2.11 Statistical analysis

The entire statistical study was performed by using R software (version 4.1.0). R software and a Perl language package were used to create the graph. The expression levels of m7G-related DEGs in malignant and non-cancerous tissues were compared using the Wilcoxon test. The relationship between genes associated with m7G and overall survival (OS) in breast cancer patients was examined using univariate Cox regression. Searching for genes associated with m7G was done using multivariate Cox regression analysis and Lasso regression. Bilateral statistical tests were conducted, and the threshold for statistical significance was set at *p* < 0.05.

## 3 Results

### 3.1 Acquisition of differential genes

**FIGURE 1:**
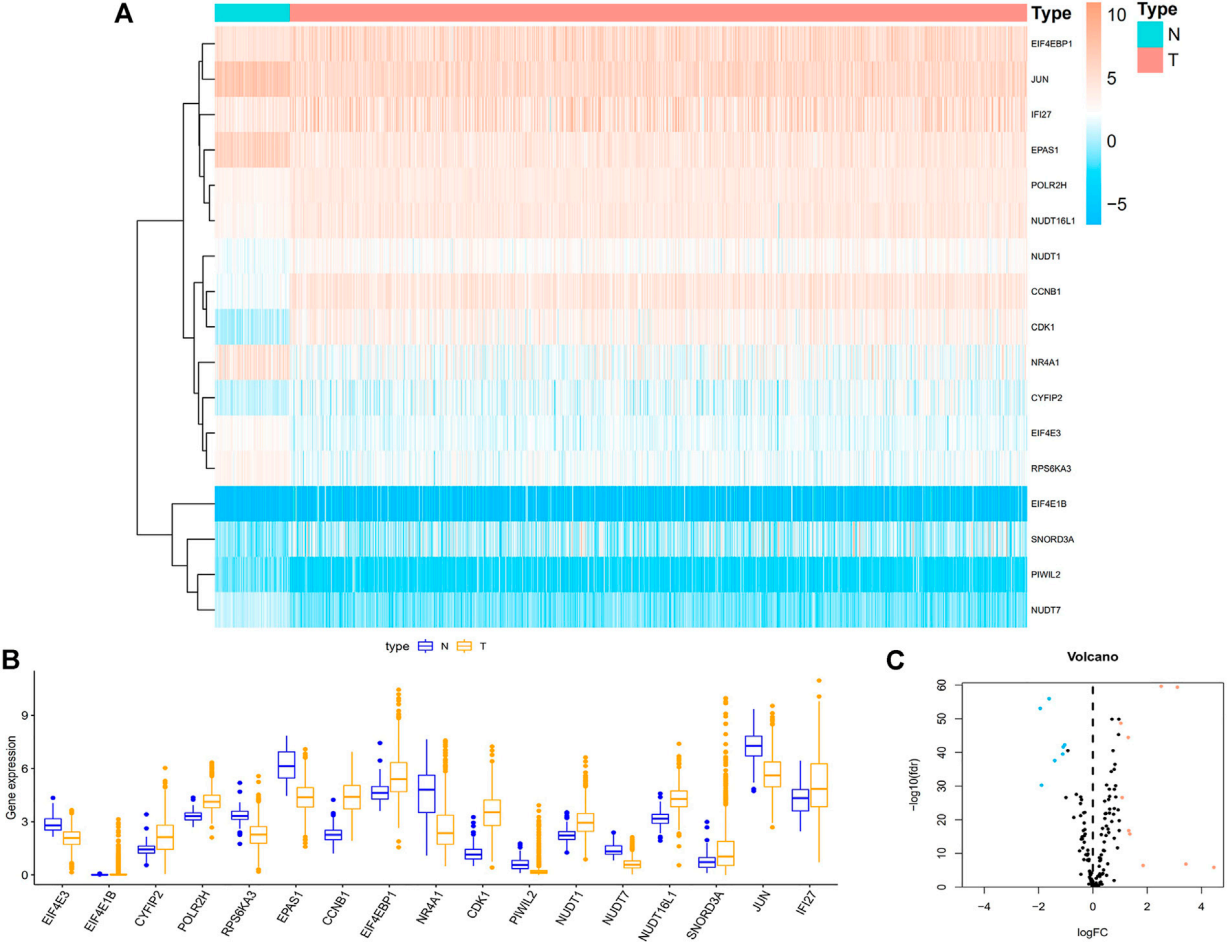
Acquisition of differentially expressed 17 m7G-related genes in BC. **(A)** Heat map of 17 m7G-related genes between BC and paracancerous tissue. **(B)** The boxplot of 17 identified m7G-related genes. Orange represents CC tissues, while purple represents normal tissues, respectively. **(C)** Volcano plot of differentially expressed m7G-related genes. Orange represents high expression, blue represents low expression, and black represents no difference between BC and normal tissues. Abbreviations: BC, breast cancer; N, normal tissue; T, tumor tissue; m7G, N7-Methylguanosine.

We obtained RNA-seq data of 1,113 breast cancer tissues and 113 normal tissues from TCGA database. The DEGs between breast cancer and non-tumor tissues were compared using the Wilcoxon test (|log2FC|>1, FDR<0.05). 17 genes associated with m7G were found, including 7 down-regulated genes and 10 up-regulated genes (Figures 1A–C).

### 3.2 Building a predictive signature for m7G-Related lncRNAs in BC

**FIGURE 2:**
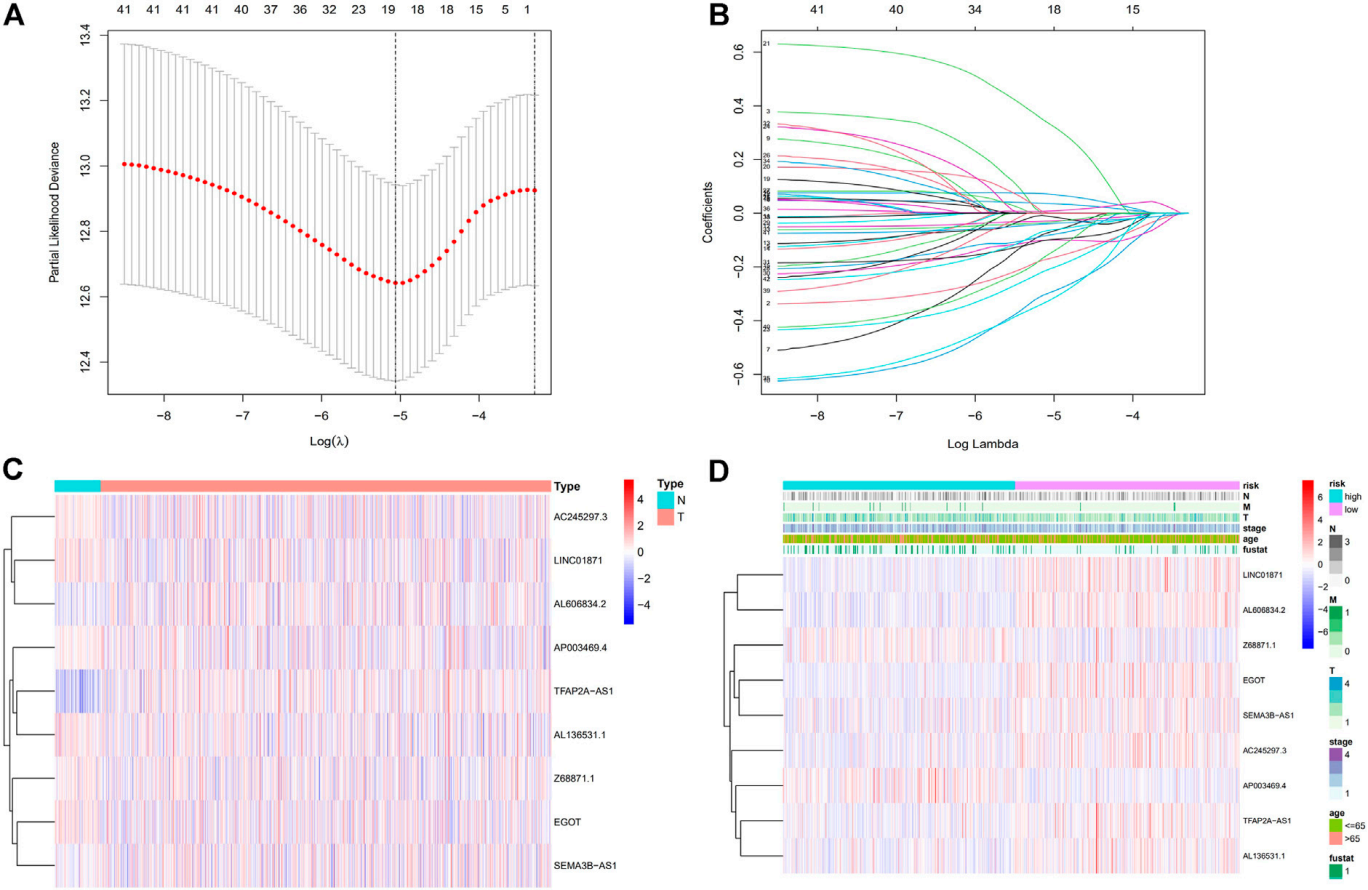
Establishment of the BC necroptosis-related lncRNA signature. **(A)** Using tenfold cross-validation, the optimal turning parameters (logλ) are determined. **(B)** The least absolute shrinkage and selection operator (LASSO) algorithm’s 10-fold cross-validation for variable selection. **(C)** The expression levels of nine m7G-related lncRNAs in BC and normal tissues. **(D)** Nine prognostic m7G-related lncRNAs and clinicopathological factors’ distribution heat maps in the high-risk and low-risk populations. Abbreviations: BC, breast cancer; m7G, N7-Methylguanosine; lncRNAs, long noncoding RNAs; N, lymph node; M, metastasis; T, tumor.

**FIGURE 3:**
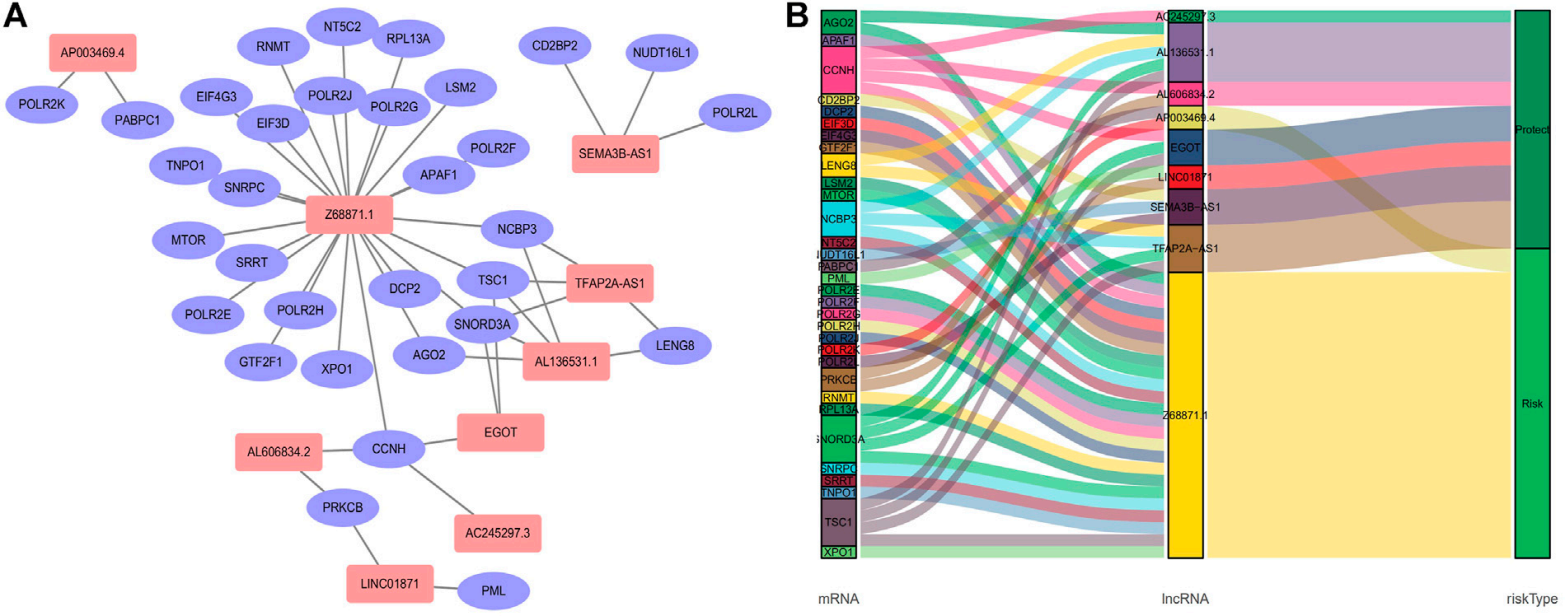
Relationship between LncRNAs and mRNAs related to N7-Methylguanosine genes. **(A)** The co-expression network of prognostic m7G-related lncRNAs. **(B)** Sankey diagram of prognostic m7G-related lncRNAs. Abbreviations:lncRNAs, long noncoding RNAs; m7G, N7-Methylguanosine; BC, breast cancer.

We obtained a total of 1,090 lncRNAs related to m7G. 43 LncRNAs were connected to the prognosis of BC patients, according to a univariate Cox regression analysis (Supplementary Figure S1). Nine lncRNAs associated with m7G (LINC01871, AP003469.4, Z68871.1, AC245297.3, EGOT, TFAP2A-AS1, AL136531.1, SEMA3B-AS1, AL606834.2) could be employed as a predictive signature in patients with BC, according to multivariate Cox regression and Lasso regression analysis (Figures 2A–C). Among them, (LINC01871, AC245297.3, EGOT, TFAP2A-AS1, AL136531.1, SEMA3B-AS1, AL606834.2) is a protective factor, and (AP003469.4, Z68871.1) is a risk factor. Additionally, the distribution of nine m7G-related lncRNAs and clinicopathological factors in high-risk and low-risk groups were also shown on a heat map (Figure 2D). The result showed that there were differences in gender (*p* < 0.001), clinical stage (*p* < 0.05), T stage (*p* < 0.05), M stage (*p* < 0.05), and N stage (*p* < 0.05) between two clusters, but did not present any differ significantly in age. Then, to display the link between LncRNAs and mRNAs, we created a network diagram of lncRNAs and mRNAs using Cytoscape (Figure 3A), and at the same time, we created a Sankey diagram (Figure 3B). The risk score for each BC patient was then determined based on the correlation coefficient determined by multivariate Cox regression analysis, and the patients were split into low-risk groups and high-risk groups based on the median risk score. The formula we used to determine the risk score is as follows: RiskScore=(−0.549×LINC01871 expression) + (0.553×AP003469.4 expression) + (0.746×Z68871.1 expression) + (−0.449×AC245297.3 expression) + (−0.458×EGOT expression) + (−0.665×TFAP2A-AS1 expression) + (−0.642×AL136531.1 expression) + (−0.270×SEMA3B-AS1 expression) + (−0.806×AL606834.2 expression).

### 3.3 N7-Methylguanosine-Related LncRNA signature and BC patients’ prognosis: A correlation

**FIGURE 4:**
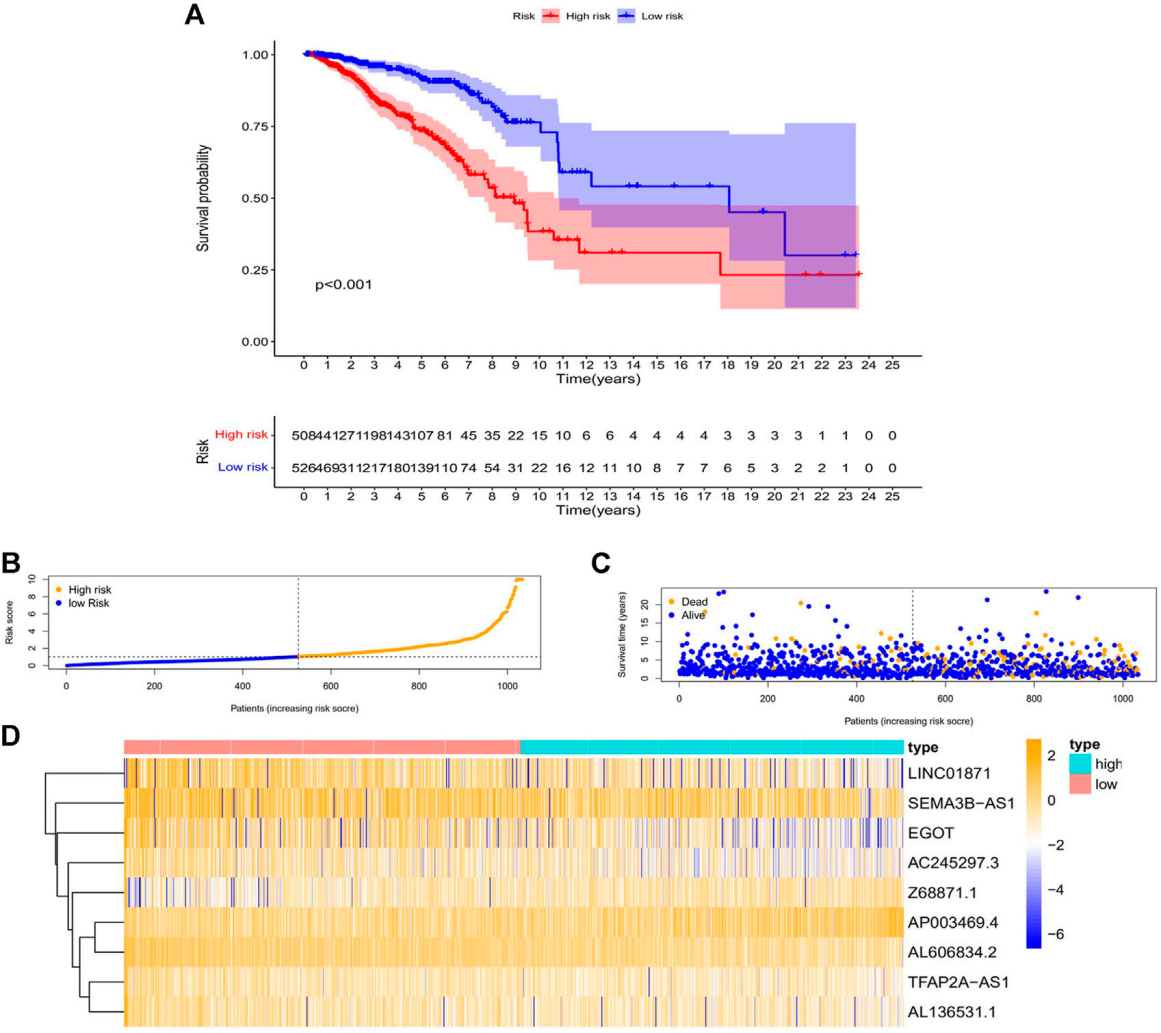
The relationship between the CC patients’ prognosis and the predicting characteristic. **(A)** Kaplan-Meier analysis compares the OS for high- and low-risk BC patients. **(B)** The risk score distribution among BC patients. **(C)** The number of patients with various risk scores that are died and alive. Orange indicates how many people died, and purple indicates how many people survived. **(D)** Expression heat map of nine lncRNAs associated with m7G. Abbreviations: m7G, N7-Methylguanosine; lncRNAs, long noncoding RNAs; BC, breast cancer; OS, overall survival.

**FIGURE 5:**
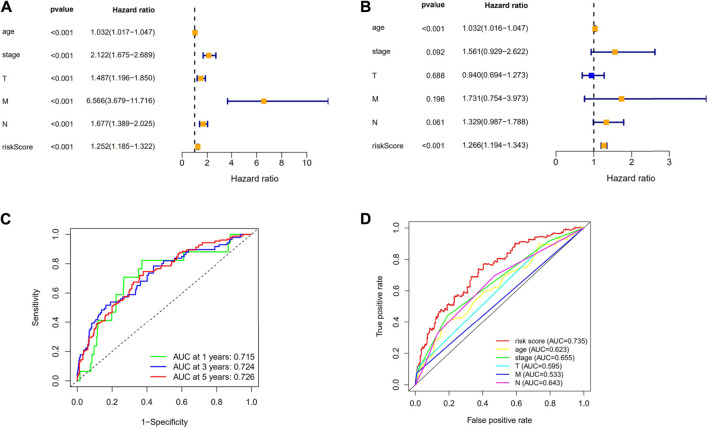
Evaluation of the necroptosis-related lncRNAs’ prognostic signature. **(A)** A univariate Cox regression analysis was performed on the clinical features and risk score. **(B)** Risk score and clinical characteristics are subjected to a multivariate Cox regression analysis. **(C)** ROC curve for forecasting the survival rate at the 1-, 3- and 5-year. **(D)** The risk score and clinicopathological factors’ ROC curves. Abbreviations: m7G, N7-Methylguanosine; lncRNAs, long noncoding RNAs; BC, breast cancer; ROC, receiver operating characteristic; AUC, area under the curve; T, tumor; N, lymph node; OS, overall survival.

**FIGURE 6:**
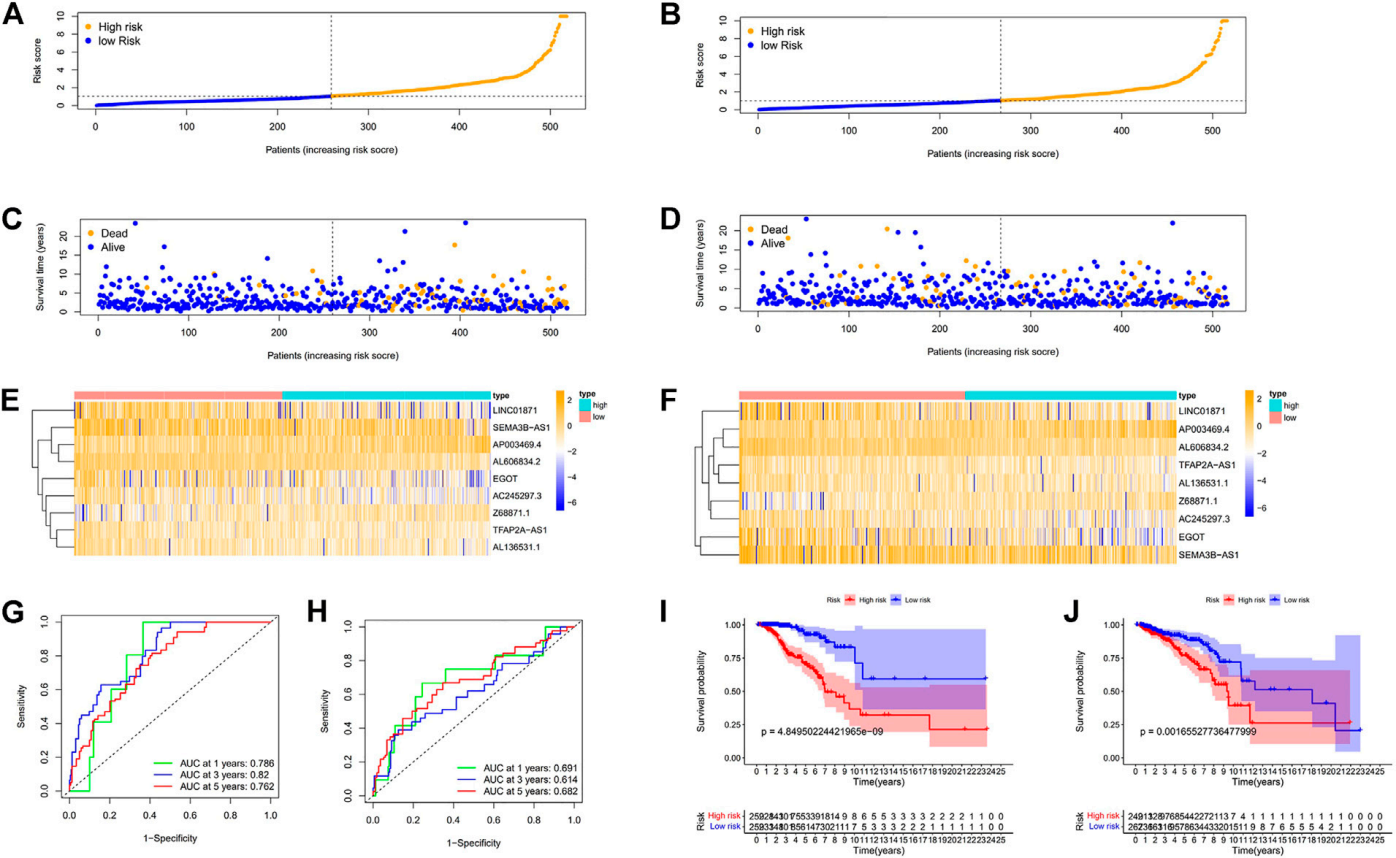
Prognosis values of the 9 m7G-related lncRNA signatures in the train, test. The risk curve **(A**,**B)**, survival scatter diagram **(C**,**D)**, heat maps of 9 lncRNA expressions **(E**,**F)**, ROC curve of BC patients **(G**,**H)**, Kaplan–Meier survival curves **(I**,**J)** between low-and high-risk groups in the train, test, respectively. Abbreviations: m7G, N7-Methylguanosine; lncRNAs, long noncoding RNAs; BC, breast cancer; ROC, receiver operating characteristic; AUC, area under the curve.

**FIGURE 7:**
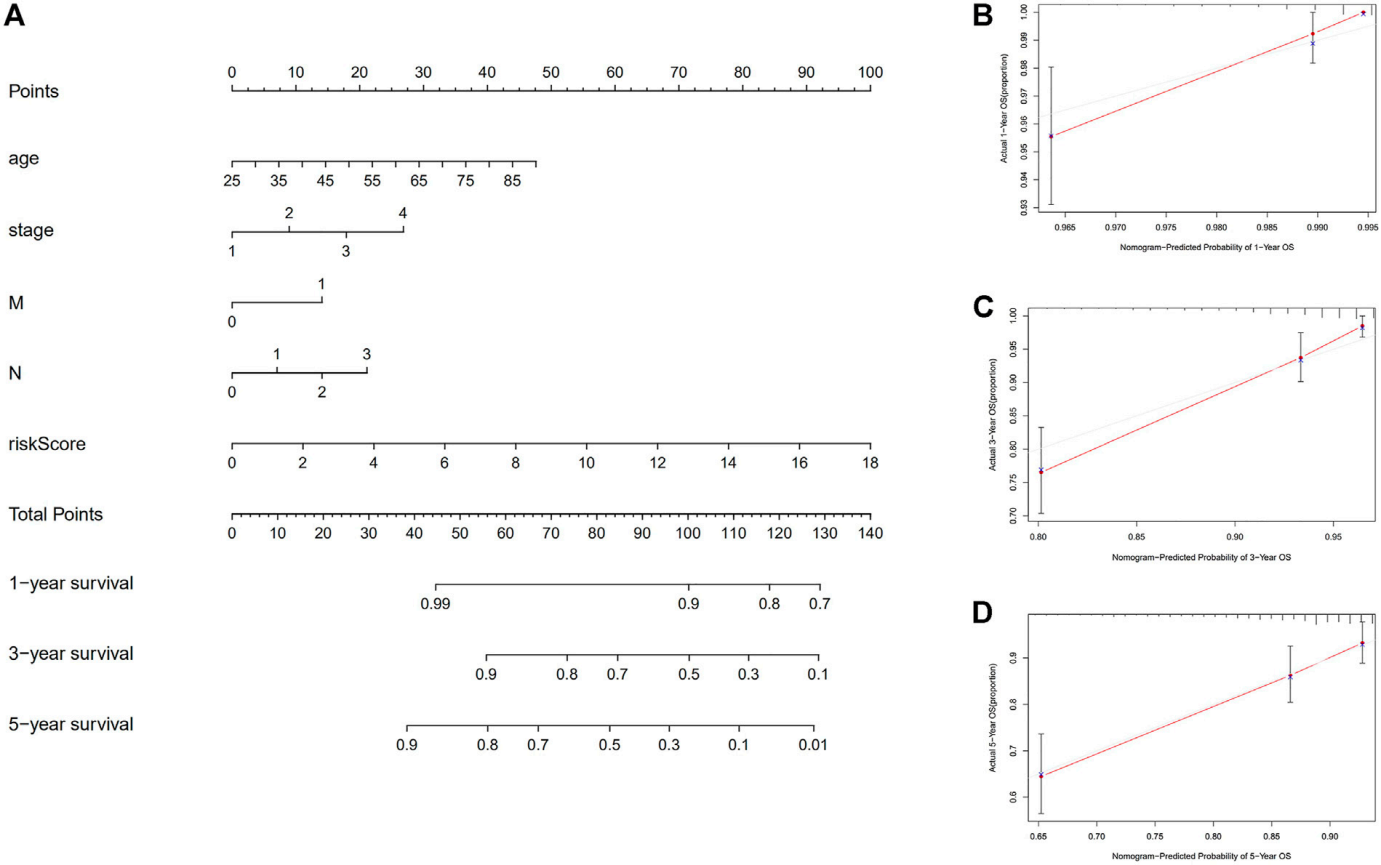
Construction of nomogram. **(A)** A nomogram that integrates clinicopathological factors and risk score forecasts the 1, 3, and 5 years of OS of BC patients. **(B**–**D)** The calibration curves examine whether the forecasted survival rates at 1, 3, and 5 years are consistent with the actual OS rates at those times. Abbreviations: OS, overall survival; BC, breast cancer.

First, to establish a connection between the m7G-related lncRNA profile and the prognosis of BC patients, we performed a Kaplan-Meier analysis to evaluate the survival durations between high-risk and low-risk BC groups. As shown in the figure (Figure 4A), the high-risk group’s OS time is significantly shorter than that of the low-risk group’s. The heat map, scatter map, and risk curve are all drawn at the same time (Figures 4B–D), and it can be seen that over time, the higher the risk score, the greater the number of BC patient deaths and the worse the prognosis. After that, we ran multivariate ROC analysis and univariate and multivariate Cox regression analysis to see if the predictive signature is a reliable predictor of prognosis in BC patients. Age, TNM stage, clinical stage, and risk score (*p* < 0.05) were substantially linked with OS in BC patients, as shown in the figure from univariate Cox regression analysis (Figure 5A). And in patients with BC, multivariate Cox regression analysis revealed that age and risk score (*p* < 0.05) were independent predictors of OS (Figure 5B). The areas under the ROC curve (AUC) values of 0.715, 0.724, and 0.726 for predicting 1-, 3-, and 5-year survival rates, respectively (Figure 5C), which demonstrate the effectiveness of the signature in predicting the prognosis of breast cancer. The risk score’s AUC value was 0.777, which was higher than that of clinical variables in predicting the prognosis of BC patients (Figure 5D). By analyzing the RiskScore, Kaplan-Meier analysis curve, risk distribution, survival outcome, and expression of lncRNAs related to necroptosis in the training set and the testing set using a single formula (Figures 6A–J), the results demonstrate the accuracy of our predictive model. Additionally, the risk score model and the clinical variables were combined to create a precise nomogram of prognosis (Figure 7A). The calibration curve demonstrates that the actual and expected survival rates at 1, 3, and 5 years have a good agreement (Figures 7B–D).

### 3.4 Relationship of the BC patients’ prognosis concerning various clinical covariates and the predictive signature

**FIGURE 8:**
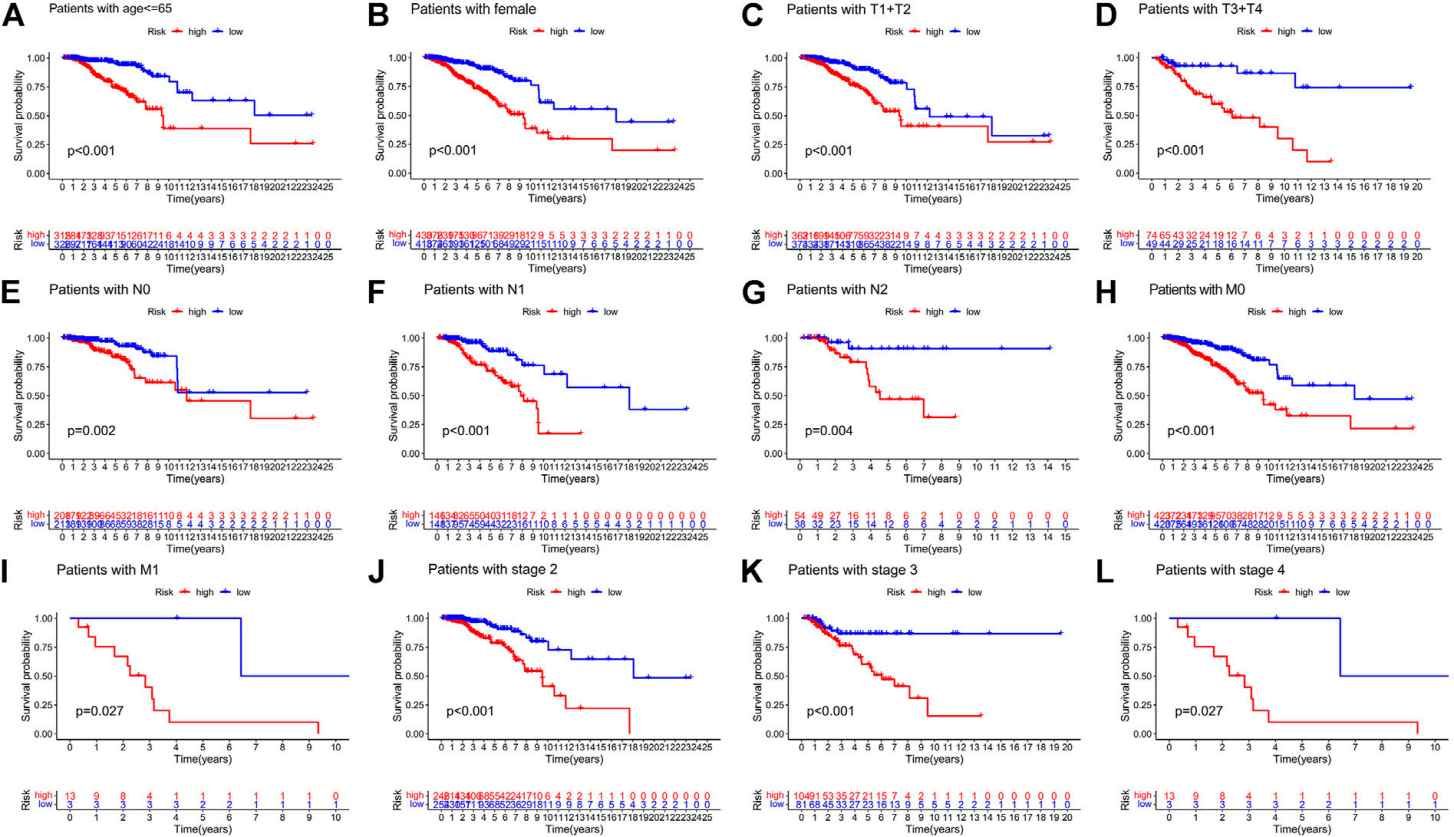
Risk curve under different clinicopathological variables. **(A)** Age. **(B)** Gender. **(C**,**D)** T stage. **(E**–**G)** N stage. **(H**,**I)** M stage. **(J**–**L)** Stage. Abbreviations: T, tumor; N, lymph node; M, metastasis.

Patients with breast cancer were separated into many groups based on clinicopathological factors to examine the impact of various clinical covariates on the prognosis of BC patients (Figures 8A–L). The OS of BC patients in the high-risk group was considerably lower than that in the low-risk group for each classification. These outcomes further demonstrated the predictive signature’s accuracy.

### 3.5 Functional analysis

**FIGURE 9:**
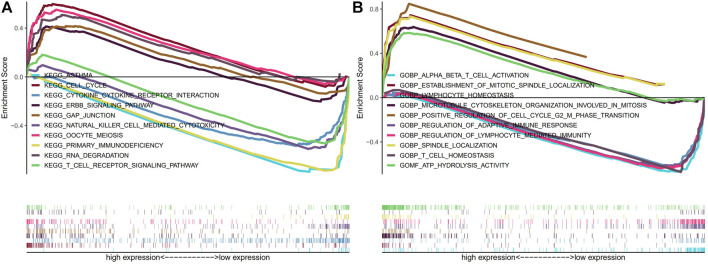
KEGG and GO enrichment analysis. **(A)** KEGG enrichment analysis. **(B)** GO enrichment analysis. Abbreviations: GO, Gene Ontology; KEGG, Kyoto Encyclopedia of Genes and Genomes.

We then carried out KEGG and GO functional enrichment analysis. In KEGG analysis (Figure 9A), the m7G-related lncRNAs were primarily clustered in the Cell cycle, Primary immunodeficiency, Erbb signaling pathway, Gap junction, RNA degradation, and Oocyte meiosis. In GO analysis (Figure 9B), the m7G-related lncRNAs were mainly concentrated in the ATP hydrolysis activity, T cell homeostasis, Spindle localization, Regulation of lymphocyte mediated immunity, Regulation of adaptive immune response, and Lymphocyte homeostasis.

### 3.6 Analysis of immune system components and activity

**FIGURE 10:**
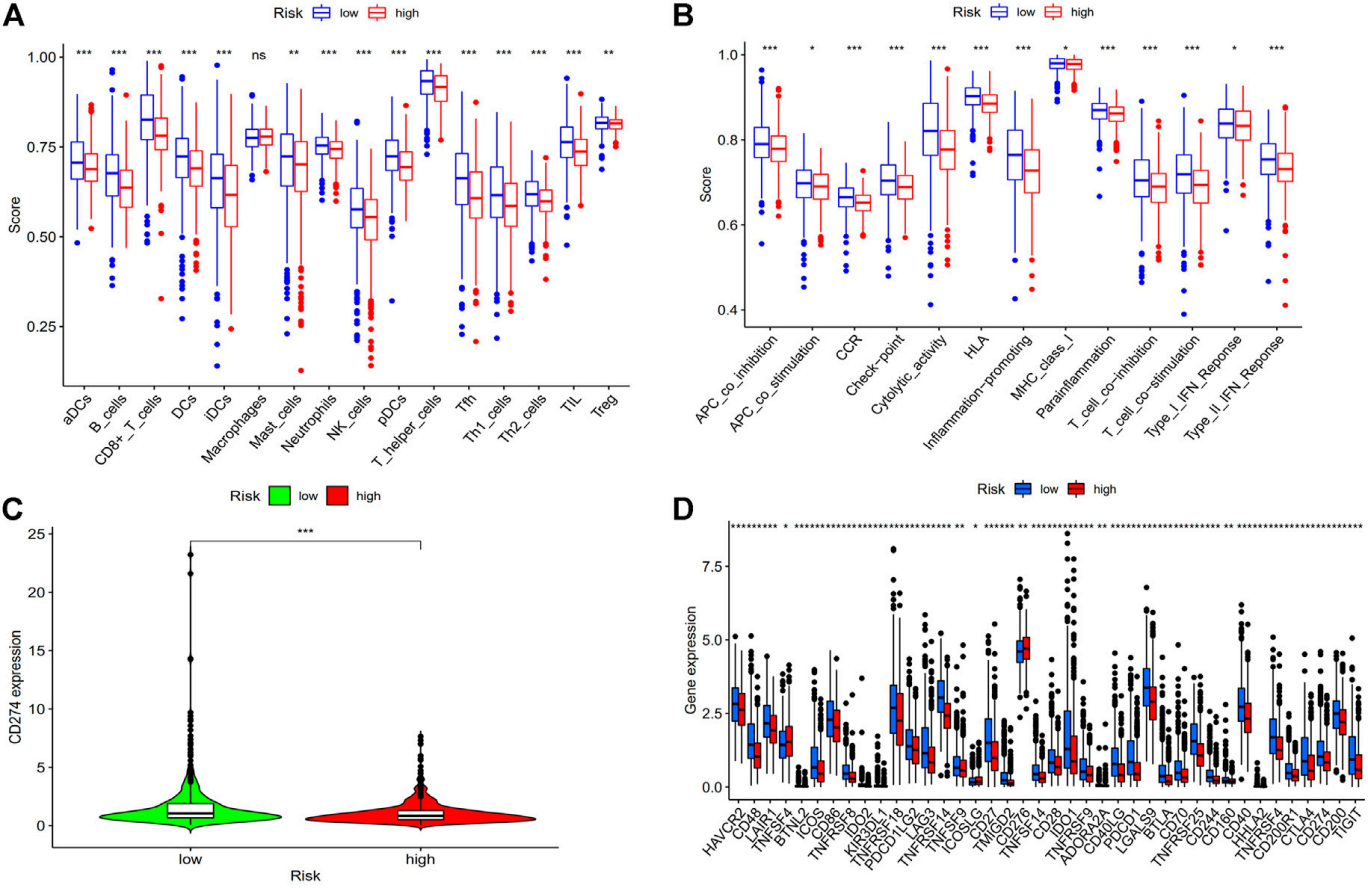
Analysis of risk score and immune related factors. **(A)** Correlation analysis of immune cells. **(B)** Analysis of immune-related pathway. **(C)** CD274 (PD-L1) expression in high- and low-risk groups. **(D)** The differences in the expression of common immunological checkpoints in the risk populations. Abbreviations: ssGSEA, single-sample gene set enrichment analysis; PD-L1, programmed cell death ligand 1; *p < 0.05; **p < 0.01; ***p < 0.001; ns, non-significant.

We looked at the relationship between the risk model and immune-related components because it has recently been discovered by researchers that immunological factors play a significant role in cancers. We analyzed immune cells and immune pathways by using ssGESA, the results showed that (Figure 10A) that aDCs, B cells, CD8^+^ T cells, NK cells, T helper cells, and Th1 cells were more expressed in the low-risk BC group. And the low-risk group had higher immune function ratings than the high-risk group for APC co-stimulation, Check−point, HLA, Inflammation−promoting, T cell co−inhibition, and Type I IFN Response (Figure 10B), which demonstrates that low-risk breast cancer patients have more active immune systems than high-risk patients. We then analyzed the immune checkpoints (Figures 10C,D), we can see from the picture nearly every immune checkpoint has been revealed to be more active in the low-risk BC group, including CD274 (PDL1), LAG3, PDCD1 (PD1), TNFRSF18 and LGALS9, suggesting that these individuals may be more responsive to immunotherapy.

### 3.7 Drug sensitivity analysis

**FIGURE 11:**
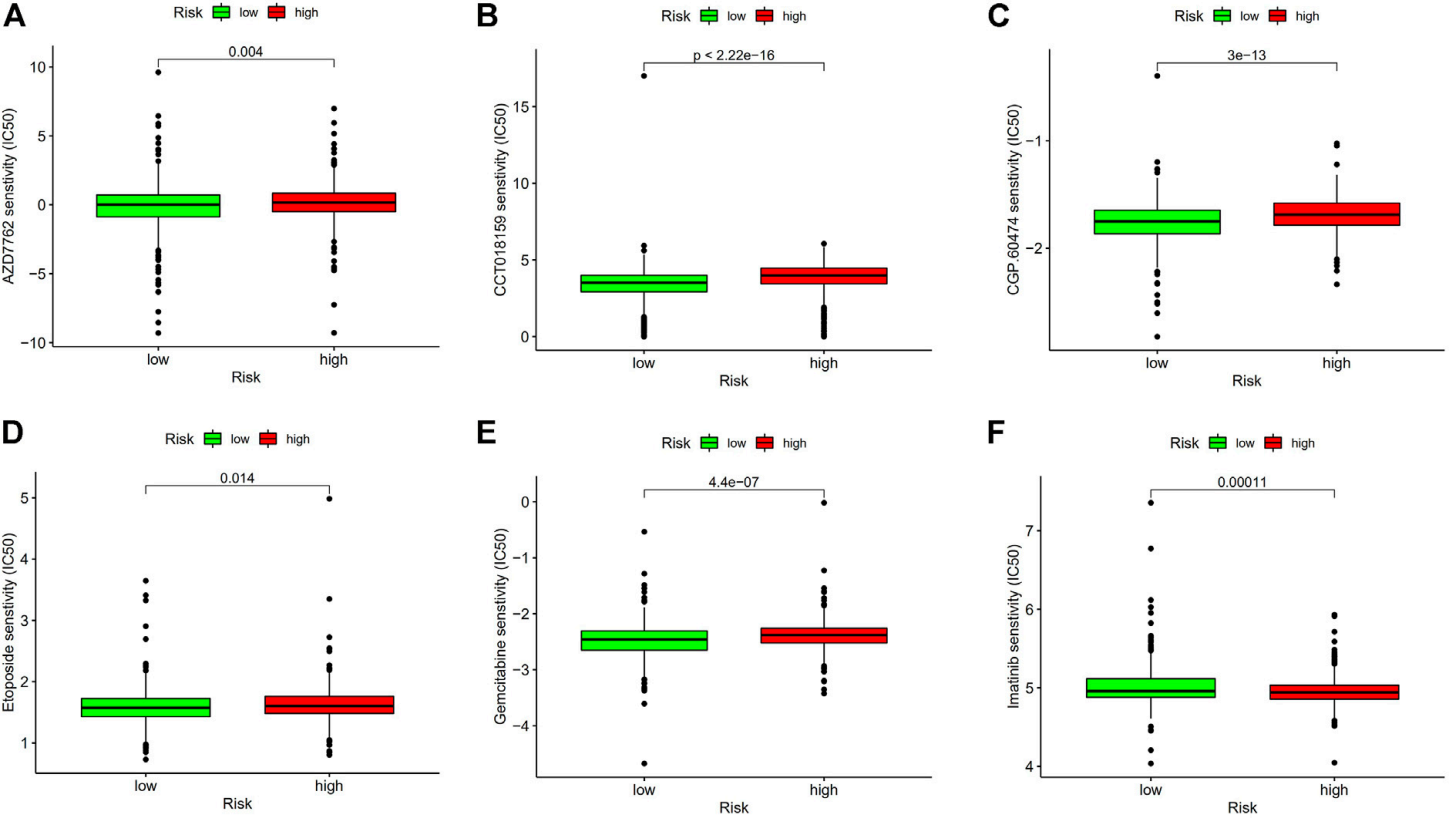
Drug sensitivity analysis. **(A)** IC50 of AZD7762 in high- and low-risk groups. **(B)** IC50 of CCT018159 in high and low-risk groups. **(C)** IC50 of CGP.60474 in high and low-risk groups. **(D)** IC50 of Etoposide in high and low-risk groups. **(E)** IC50 of Gemcitabine in high and low-risk groups. **(F)** IC50 of Imatinib in high- and low-risk groups. Abbreviations: IC50, half-maximal inhibitory concentration.

Since low-risk BC patients are more responsive to immunotherapy, we contrasted the medication sensitivity between groups at high-risk and low-risk (Figures 11A–F), the findings revealed that low-risk BC patients were more responsive to immune drugs AZD7762, CCT018159, CGP.60474 and chemotherapeutic drug Etoposide, and Gemcitabine, but resistant to targeted drug Imatinib. These results prove that investigating specialized treatment plans appropriate for BC groups with high and low risk is beneficial.

### 3.8 The reliability of the m7G-related lncRNA signature is further demonstrated by principal component analysis

**FIGURE 12:**
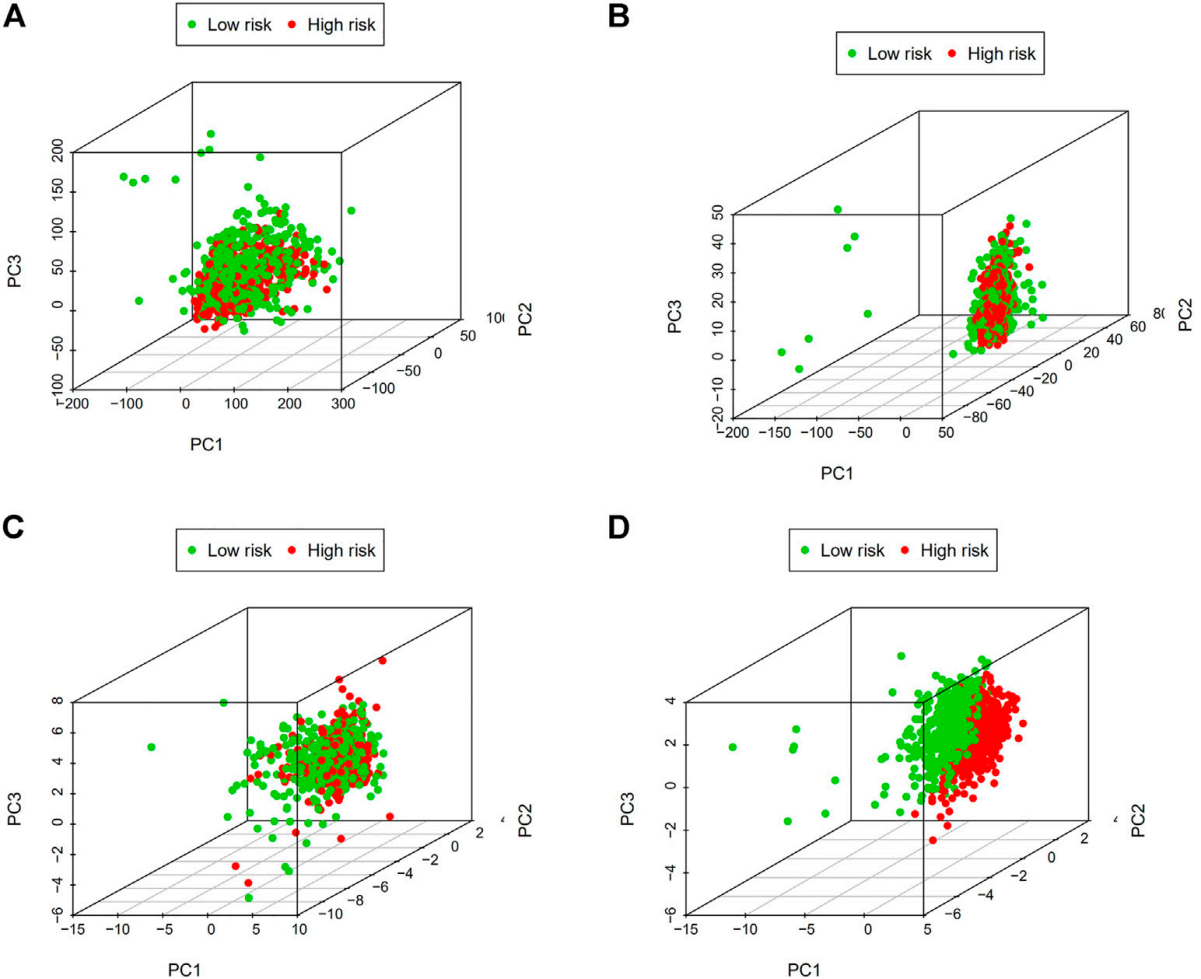
Principal component analysis (PCA). **(A)** The whole gene expression profiles. **(B)** Genes related to m7G. **(C)** LncRNAs connected to m7G. **(D)** Predictive signature based on lncRNAs associated with m7G. Abbreviations: m7G, N7-Methylguanosine. lncRNAs; long noncoding RNAs.

It was confirmed that there was a difference between high-risk and low-risk BC groups using principal component analysis (PCA). The picture shows (Figures 12A–D) that the prognostic risk model can distinguish between high-risk and low-risk BC groups, which further illustrates the accuracy of the signature.

### 3.9 Correlation analysis between predictive model and clinical variables

**FIGURE 13:**
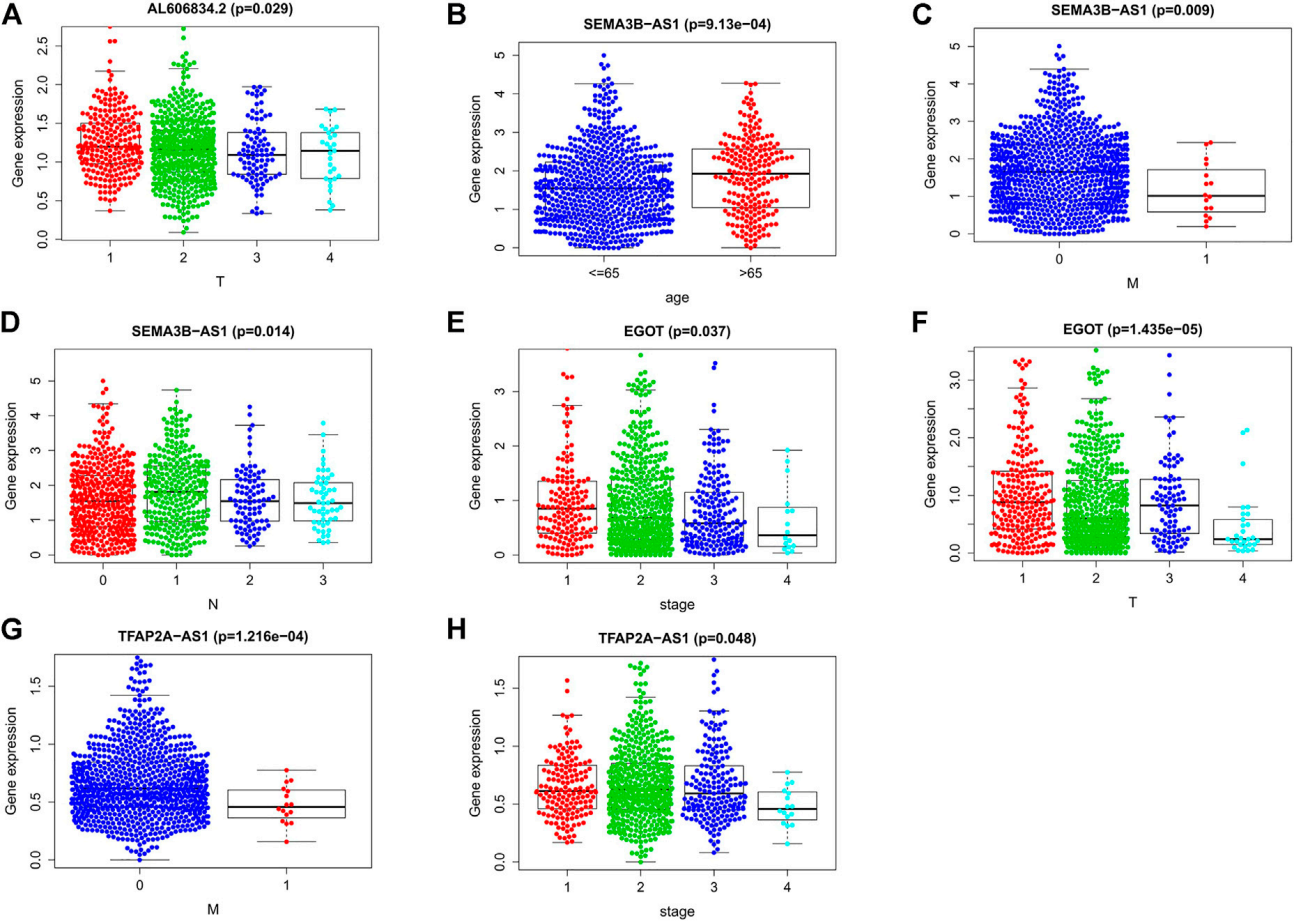
Connections between the nine lncRNAs associated to m7G and clinical traits. **(A)** Association between lncRNA AL606834.2 expression and stage T. **(B**–**D)** Association between lncRNA SEMA3B-AS1 expression level and age, stage M, stage N. **(E**,**F)** Association between lncRNA EGOT expression level and clinical stage, stage T. **(G**,**H)** Association between lncRNA TFAP2A-AS1 expression level and clinical stage, stage M. Abbreviations: m7G, N7-Methylguanosine; lncRNAs, long noncoding RNAs; N, lymph node; M, metastasis; T, tumor.

We examined the relationship between these clinical characteristics and the predictive signature using gene expression data and related clinical data from the TCGA database (Figures 13A–H): LncRNA AL606834.2 is related to stage T; LncRNA EGOT is related to clinical stage and stage T; LncRNA SEMA3B-AS1 is related to age, stage M, and stage N; LncRNA TFAP2A-AS1 is related to clinical stage and stage M.

### 3.10 External verification of the expression of m7G-related lncRNAs in BC

**FIGURE 14:**
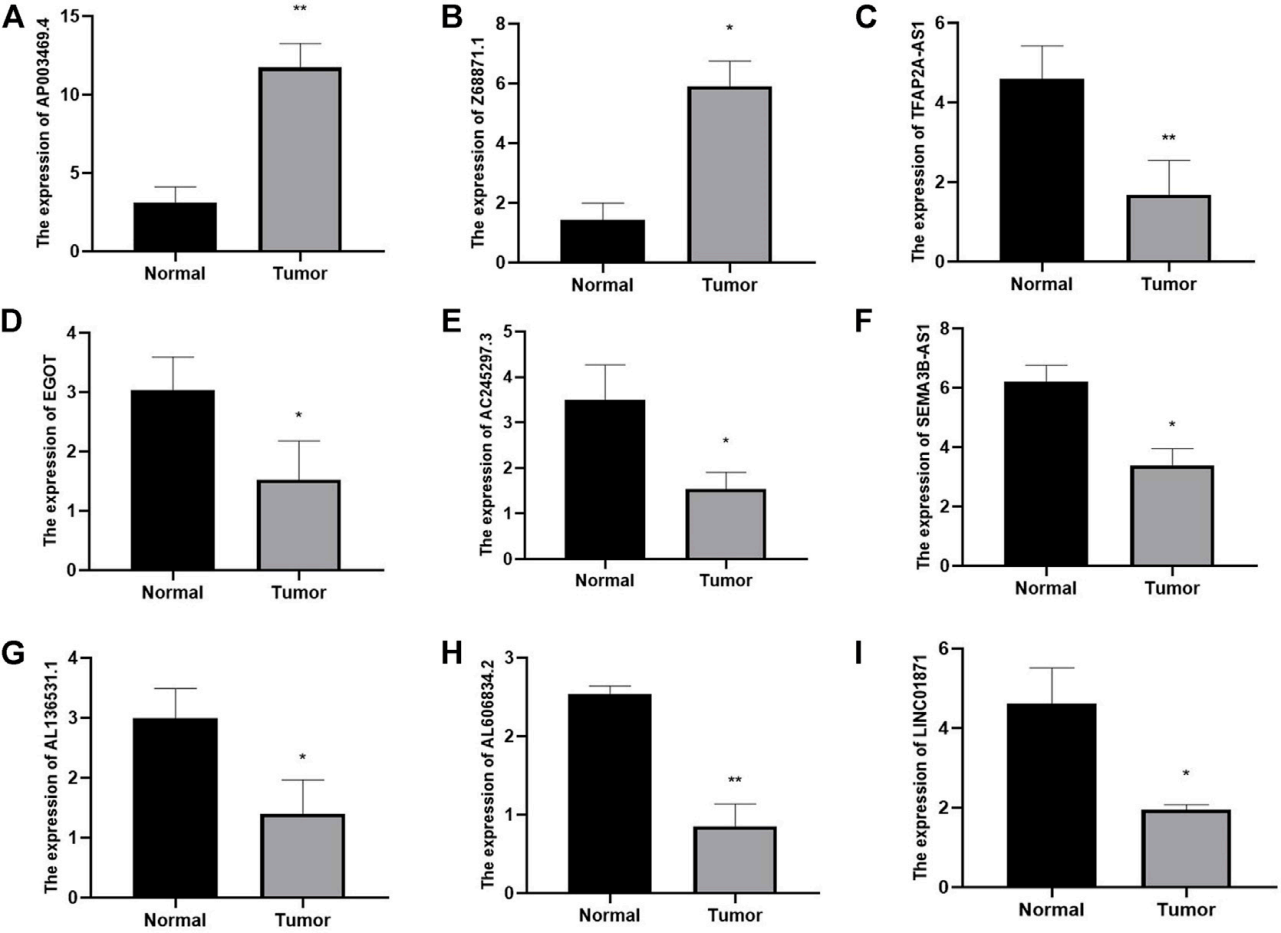
Identifying plasma lncRNAs linked to m7G in breast cancer was done using qRT-PCR analysis. The relative expression levels of plasma AP003469.4 **(A)**, Z68871.1 **(B)**, TFAP2A-AS1 **(C)**, EGOT **(D)**, AC245297.3 **(E)**, SEMA3B-AS1 **(F)**, AL136531.1 **(G)**, AL606834.2 **(H)**, LINC01871 **(I)** were measured in breast cancer tissues and normal tissues by qRT-PCR. *p < 0.05.**p < 0.01.

By using qRT-PCR, we detected the relative expression levels of the 9 m7G-related lncRNAs in BC tissues and normal tissues. The findings showed that AP003469.2 and Z68871.1 considerably increased in BC tissues while LINC01871, AC245297.3, EGOT, TFAP2A-AS1, AL136531.1, SEMA3B-AS1, and AL606834.2 significantly decreased (Figure 14). These outcomes were in line with those of the database analysis.

## 4 Discussion

The most prevalent malignant tumor in women worldwide and one of the leading causes of cancer death is breast cancer. Fortunately, the death rate of breast cancer has been significantly lowered thus far as a consequence of advancements in detection and treatment over the past several years, but it is still unable to produce satisfactory outcomes (Shen et al., 2020). Therefore, to accurately estimate the prognosis of BC patients, it is crucial to identify a trustworthy biomarker. N7-Methylguanosine (m7G) plays a significant role in the incidence and progression of cancers by modulating the expression of numerous oncogenes and tumor suppressor genes, as has been discovered recently in an increasing number of studies (Luo et al., 2022). Xin-Yu Li et al. constructed a prognostic risk model of renal cell carcinoma based on N7-methyladenosine (m7G) methylation regulatory factors (Ming and Wang, 2022). The development of the breast cancer predictive model based on m7G-related LncRNAs, however, has not been studied.

By using the Cancer Genome Atlas (TCGA) database, we established a cohort of 1,113 breast cancer tissue samples and 113 normal tissue samples and screened lncRNAs associated with m7G by constructing a co-expression network of lncRNAs and m7G-related genes. Lasso regression analysis and Cox regression analysis were used to identify nine lncRNAs linked to m7G (LINC01871, AP003469.4, Z68871.1, AC245297.3, EGOT, TFAP2A-AS1, AL136531.1, SEMA3B-AS1, AL606834.2). The nine m7G-related lncRNAs could be used as the prognostic signature and therapeutic targets in breast cancer patients. Seven related LncRNAs (LINC01871, AP003469.4, Z68871.1, AC245297.3, EGOT, TFAP2A-AS1, SEMA3B-AS1) were reported to be associated with cancer. By constructing the co-expression network of autophagy-related mRNA-lncRNA from the Cancer Genome Atlas (TCGA) database, Qianxue Wu et al. demonstrated that (Wu et al., 2021) lncRNA LINC01871 may be a predictive marker of autophagy in breast cancer and is advantageous to the prognosis of patients with BC. According to Shigui Tao et al. (Tao et al., 2022), lncRNA LINC01871 is a necroptosis-related lncRNA, which may reliably predict the prognosis of breast cancer. By creating the AP003469.4-miRNAs-mRNAs ceRNA network, Tengyang Fan et al. found that lncRNA AP003469.4 may be a potential biomarker of hepatocellular cancer (Fan et al., 2022). Wenchang Lv et al. came to the conclusion that lncRNA (Z68871.1, EGOT) is an independent prognostic factor and a predictive signature of m6A-related lncRNA in breast cancer (Lv et al., 2021). Xiaoying Li et al. pointed out that (Li et al., 2020) lncRNA AC245297.3 plays a role in the dry regulation of breast cancer stem cells (BCSC) and is closely linked to breast cancer patient’s prognosis. By using bioinformatics research, Guo Jie et al. identified the role of lncRNA TFAP2A-AS1 in cell proliferation and examined its expression and function using RT-qPCR and MTS. It is made clear that oral squamous cell carcinoma (OSCC) cells can multiply, migrate, and invade by overexpressing the long noncoding RNA (lncRNA) TFAP2A-AS1 (Jie et al., 2022). MiR-718 mediates the indirect interaction between the lncRNA SEMA3B-AS1 and PTEN to control the proliferation of hepatocellular carcinoma cells, as demonstrated by Yuchuan Zhong et al. (Zhong et al., 2019). ZhimingDong et al. have shown that (Dong et al., 2019) the down-regulation of tumor suppressor genes SEMA3B and lncRNA SEMA3B-AS1 mediated by hypermethylation of promoter is related to the progression and prognosis of esophageal squamous cell carcinoma. It is unknown how the two additional lncRNAs (AL136531.1 and AL606834.2) related to m7G affect the prognosis of cancer patients. Therefore, more research is required to determine how these lncRNAs impact the prognosis of BC patients. Patients with BC were then separated into low-risk and high-risk groups based on the median. The outcomes demonstrated that the risk score was an accurate predictor of the prognosis for BC patients, and the low-risk group had a better prognosis than the high-risk group. Then, using expected outcomes as a guide, we created a nomogram to estimate the prognosis of BC patients. The risk score has the ability to predict survival and can independently predict the prognostic risk of BC, according to the areas under the ROC curve (AUC) for predicting 1-, 3-, and 5-year survival rates, which were 0.715, 0.724, and 0.726, respectively. The predictive signature has excellent predictive performance, according to internal verification. Finally, we used qRT-PCR for external verification, and the results further proved the accuracy of our signature.

We used KEGG and GO functional enrichment analysis of these m7G-related LncRNAs to investigate the potential biological roles of our signature. The m7G-related lncRNAs were mostly concentrated in the Cell cycle, Primary immunodeficiency, Erbb signaling pathway, Gap junction, RNA degradation, and Oocyte meiosis, according to KEGG analysis. In GO analysis, the m7G-related lncRNAs were mainly concentrated in the ATP hydrolysis activity, T cell homeostasis, Spindle localization, Regulation of lymphocyte-mediated immunity, Regulation of adaptive immune response, and Lymphocyte homeostasis. To better understand the potential relationship between the Erbb signaling pathway and miR-34a, Yilin Wang et al. used luciferase reporter gene analysis (Wang et al., 2017), and they discovered that overexpressing miR-34a decreased the expression of Erbb2 and prevented the invasion and growth of breast cancer cells *in vitro*. Based on the bioinformatics analysis of prospective biomarkers for breast cancer with poor prognoses, Gang Chen et al. identified 11 hub genes that were substantially correlated with BC patient prognoses and investigated the interactions between these hub genes and the KEGG Cell cycle using GSEA (Chen G. et al., 2021). Triple negative breast cancer (TNBC) cells were shown to have a high concentration of stem cell-like traits, and Leticia Serrano-Oviedo et al. discovered that the dry gene Gap Junction Protein Alpha 1 (GJA1) contributes to a bad prognosis in TNBC patients (Serrano-Oviedo et al., 2020). T cell and lymphocyte homeostasis have also been shown to be crucial in carcinogenesis and development (Takaki et al., 2017; Koukourakis and Giatromanolaki, 2021). Interestingly, GESA analysis shows that immune-related pathways are enriched in the low-risk group, which also provides a theoretical basis for our next ssGSEA analysis.

Given the tight association between m7G and immune-mediated pathways, we used ssGSEA to investigate the connection between immune cell subsets and related functions using TCGA-BRCA data. The results show that low-risk BC patients have higher expression of aDCs, B cells, CD8^+^ T cells, NK cells, and immune function scores of APC co inhibition, Checkpoint, Inflammationpromoting, T cell coinhibition, Type I IFN Response were higher in the low-risk groups than in the high-risk groups. Studies have demonstrated a correlation between improved clinical outcomes for breast cancer and greater CD8^+^ T and CD4^+^ T cell infiltration in tumors (Jin and Hu, 2020). It has been demonstrated by Rossana Tallerico et al. that NK cells can prevent the hematological spread of breast cancer and associated cancer stem cells, which is advantageous for the prognosis of breast cancer patients (Tallerico et al., 2017). By using immunohistochemical staining, RT-PCR, and Western blotting to identify the related molecules of IL-12/signal transducer and activator of transcription 4 (STAT4), Xin Zhao et al. investigated the mechanism of SaikosaponinA (SSa). The results revealed that, in comparison to the control group, SSa significantly reduced tumor growth and tumor cell proliferation and increased anti-tumor immunity, which reduced breast cancer growth by shifting the balance of Th1/Th2 to Th1, as seen by the rise in infiltrating CD8+T cells and CD4+T cells in the tumor (Zhao et al., 2019). CaseyD.Stefanski et al. showed that adriamycin resistance in breast cancer cells results from the loss of APC through a diminished response to DNA damage, while the inhibitory effect of DNA damage can be blunted by the inhibitory effect of DNA repair (Stefanski et al., 2019). The lack of type I IFN response reduces the antitumor activity of natural killer cells in breast cancer, speeding up the disease’s progression, as demonstrated by DamienJ Zanker et al. (Zanker et al., 2021). These findings also provide an explanation for why these immune subsets and immunological-related processes are more active in low-risk BC patients. Therefore, the poor prognosis in the high-risk BC group may be brought on by insufficient antitumor immune function. Last but not least, we also performed a study of immune checkpoint expression and medication sensitivity; the results revealed that immune checkpoint expression increased in the majority of low-risk BC patients, suggesting that low-risk BC patients may be more responsive to PD-1/L1 immunotherapy. Meanwhile, combining immunotherapy with chemotherapy may be beneficial for low-risk BC patients, according to the drug sensitivity analysis that revealed they were more sensitive to immune drug AZD7762, CCT018159, CGP.60474 and chemotherapeutic drug Etoposide, Gemcitabine but resistant to the targeted drug imatinib. This information was crucial for clinical guidance and provided proof for an accurate and individualized treatment of BC patients.

The study has a number of limitations. In the beginning, we only use the TCGA database for internal verification, which may lead to errors due to the small amount of data, therefore we require additional data sets to confirm our research. Secondly, we have not studied the effect of the signature on the prognosis of different breast cancer subtypes, and we hope to conduct more in-depth research in this area in the future.

## Data Availability

The original contributions presented in the study are included in the article/Supplementary Material, further inquiries can be directed to the corresponding author.

## Supplementary material

Supplementary Figure S1 43 m7G-related lncRNAs were associated with the prognosis of BC patients by univariate cox regression analysis.
