# Supplementary material for: Construction of prognostic signature of breast cancer based on N7-Methylguanosine-Related LncRNAs and prediction of immune response
Source: Front Genet. 2022 Oct 24;13:991162. doi: 10.3389/fgene.2022.991162 (PMC9639662; doi:10.3389/fgene.2022.991162)
Supplement: Supplementary file 2 [file Table2.DOCX]

| Variables |  |  |  |  |  |  |  |  |  | TCGA dataset (n = 1041) |
| --- | --- | --- | --- | --- | --- | --- | --- | --- | --- | --- |
| Age |  |  |  |  |  |  |  |  |  |  |
| ≤65 |  |  |  |  |  |  |  |  |  | 749 |
| >65 |  |  |  |  |  |  |  |  |  | 292 |
| Gender |  |  |  |  |  |  |  |  |  |  |
| Female |  |  |  |  |  |  |  |  |  | 1029 |
| Male |  |  |  |  |  |  |  |  |  | 12 |
| Stage |  |  |  |  |  |  |  |  |  |  |
| I + II |  |  |  |  |  |  |  |  |  | 769 |
| III + IV |  |  |  |  |  |  |  |  |  | 249 |
| Unknow |  |  |  |  |  |  |  |  |  | 23 |
| T |  |  |  |  |  |  |  |  |  |  |
| T1+T2 |  |  |  |  |  |  |  |  |  | 873 |
| T3+T4 |  |  |  |  |  |  |  |  |  | 165 |
| TX+Unknow |  |  |  |  |  |  |  |  |  | 3 |
| M |  |  |  |  |  |  |  |  |  |  |
| M0 |  |  |  |  |  |  |  |  |  | 862 |
| M1 |  |  |  |  |  |  |  |  |  | 21 |
| MX+Unknow |  |  |  |  |  |  |  |  |  | 158 |
| N |  |  |  |  |  |  |  |  |  |  |
| N0 |  |  |  |  |  |  |  |  |  | 486 |
| N1 |  |  |  |  |  |  |  |  |  | 355 |
| N2+N3 |  |  |  |  |  |  |  |  |  | 183 |
| NX+Unknow |  |  |  |  |  |  |  |  |  | 17 |

**Table S1** **The clinicopathological features of BC patients in this study**
