# Supplementary material for: Construction of prognostic signature of breast cancer based on N7-Methylguanosine-Related LncRNAs and prediction of immune response
Source: Front Genet. 2022 Oct 24;13:991162. doi: 10.3389/fgene.2022.991162 (PMC9639662; doi:10.3389/fgene.2022.991162)
Supplement: Supplementary file 3 [file Table3.DOCX]

**Table S3.** **Primers used in this study**

| **gene** | **primer-F (5'-3')** | **primer-R (5'-3')** |
| --- | --- | --- |
| LINC01871 | AGAACACAGACACGCCAGGGG | ACGCTGGGCTTGTGTGAGGG |
| AC245297.3 | CCACGCCCGGCCGAAAATAC | AAAAGGAATGCTTCTGAGTGCTGT |
| EGOT | CTAGCAACAGACTTCACCTG | CTTACCATCGTGCCAATCC |
| TFAP2A-AS1 | TTCTTTAATCAGGATAGGGCAC | GGTTTCCCTACTTTGAAGCTC |
| AL136531.1 | AAACCCTGGTGGTTGGTGTC | TGGCTCCTATTGTCCCTCGT |
| SEMA3B-AS1 | ATCTCAACCTCTCCCTCCA | TCATTCCCAACTGAGGCTC |
| AL606834.2 | CGTGGTAATCTGAGTGTGGTAAGC | GACCGATATGCCTATGCGAATGT |
| AP003469.4 | GGATTTCAGCGGCACAAGG | CCTGGAGATGCATACTCACACACA |
| Z68871.1 | TGGCCTGCTGTCTCCTGAAG | TGCAGACCAGTGAAGAGCCA |
