# Supplementary figures and images for: Construction of prognostic signature of breast cancer based on N7-Methylguanosine-Related LncRNAs and prediction of immune response
Source: Front Genet. 2022 Oct 24;13:991162. doi: 10.3389/fgene.2022.991162 (PMC9639662; doi:10.3389/fgene.2022.991162)

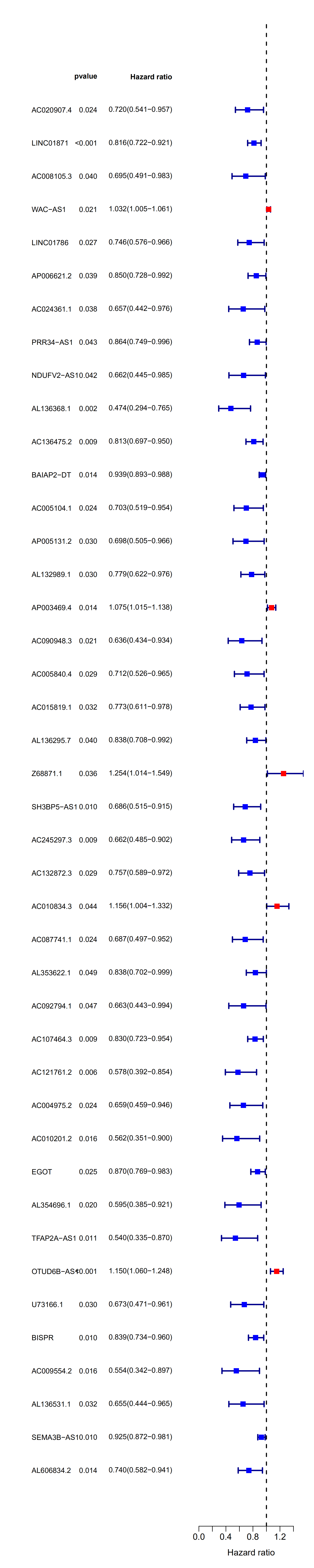

Supplement: Supplementary file 4 [file Image1.PNG]
